# Multilevel multivariate analysis on the anthropometric indicators of under-five children in Ethiopia: EMDHS 2019

**DOI:** 10.1186/s12887-022-03172-x

**Published:** 2022-03-30

**Authors:** Lijalem Melie Tesfaw, Zelalem G. Dessie

**Affiliations:** 1 Department of Statistics, Bahir Dar University, Bahir Dar, Ethiopia; 2 School of Mathematics, Statistics and Computer Science, University of KwaZulu-Natal, Durban, South Africa

**Keywords:** Multilevel multivariate logistic regression model, Stunting, Underweight, Wasting, Ethiopia, Underfive children

## Abstract

**Background:**

Undernutrition is the main cause of morbidity and mortality of children aged under five and it is an important indicator of countries’ economic and health status. Limited attention is given to research papers conducted in Ethiopia that identified and estimates the determinants of under-five anthropometric indicators by considering their association and clustering effect. Therefore, this study aimed to identify and estimate the effects of important determinants of anthropometric indicators by taking into account their association and cluster effects.

**Methods:**

In this study, a cross-sectional study design was implemented based on the data obtained from the 2019 Ethiopia Mini Demographic and Health Survey (EMDHS) consists a total of 5027 under-five children. A multilevel multivariate logistic regression model was employed to estimate the effect of the determinants given their association of anthropometric indicators and clustering effect.

**Results:**

Among 5027 children considered in the study 36.0, 23.3, and 9.1% of them were stunted, underweight, and wasted, respectively. Whereas the total number of undernourished (stunting, underweight and/or wasting) children was 42.9%. More than half of the children (51.2%) were males and 77.0% lived in rural area. The estimated odds of children from households with secondary and above education levels being stunted was 0.496 (OR = 0.496) times the estimated odds of children from households with no education. Whereas children from the richest households were less likely to be stunted as compared to children from the poorest households (OR = 0.485). The estimated odds of children from urban areas being underweight and wasting were lower by 24.9 and 33.7% of estimated odds of children from rural areas respectively.

**Conclusion:**

The prevalence of anthropometric indicators of stunting, underweight, and wasting in Ethiopia was increased. The children underweight has significant dependency with both stunting and wasting. The sex of the child, wealth index, and education level of a household are the common important determinants of stunting, underweight and wasting. The undernourished status of children was more alike within the region and differences between regions.

## Background

Undernutrition is the main cause of morbidity and mortality of children aged under five and it is an important indicator of countries’ economic and health status [1]. According to World Health Organization (WHO) report, globally, the distribution of child death and malnutrition have a substantial unequal [2]. It is most common and prevalent in low and middle-income countries such as Africa in particular Ethiopia. Though malnutrition is a relative or absolute deficiency or excess of one or more essential nutrients in our body, malnutrition due to deficiency of nutrients (undernutrition) is the most common that occurred in low and middle-income countries. Globally, undernutrition contributes to 45% of all death among under-five children [3]. In Africa, over one third of under five children (39%) [3], and in sub-Saharan Africa 41% of under five children are undernourished [4]. Ethiopia is among those countries with the highest rate of undernutrition in sub-Saharan Africa. Based on the 2016 Ethiopian Demographic and Health Survey (EDHS), the overall prevalence of undernutrition was 46.7% [3]. This leads to poor intelligence, academic and behavior performance, and delay in mental and physical developments of children [3, 4].

Undernutrition is often assessed through anthropometric indicators that involve wasting (weight-for-height), stunting (height-for-age) and underweight (weight-for-age) [2]. These anthropometric indicators are used to measure nutritional insufficiency or imbalance, causes of multifaceted health problem and in general undernutrition in a population of children. Based on 2006 WHO child growth standards, a child with calculated height-forage z-score (HAZ), weight-for-height z-score (WHZ), and weight for age z-score (WAZ) values below two are defined as having stunting, wasting and underweight respectively [2, 5].

Based on 2018 WHO global reports, the prevalence of anthropometric indicators stunting, underweight and wasting in under five children were 24.7%, 15.1%, and 7.8%, respectively [6, 7]. Even if these problems are common all over the world, it is most dominant in low and middle income countries like Ethiopia. According to the 2016 EDHS reports, in Ethiopia, the weighted prevalence of stunting, underweight, and wasting were 38.3%, 23.3%, and 10.1 %, respectively [8]. While in 2017, a community based cross-sectional study conducted in Northeast Ethiopia showed that the prevalence of stunting, underweight, and wasting was 43.1%, 24.8 %, and 16.2% respectively [6]. This is one of the implications that testifies Ethiopia shares the higher proportion of undernutrition among under five children.

The likelihood of occurrence of anthropometric indicators is associated with socio-economic, demographic, and biological factors [4]. A study conducted at Nakaseke and Nakasongola districts of Uganda reported that maternal occupation and age groups among under five children are significantly associated with malnutrition [4]. Child undernutrition is the cause of other commodities. A case control study in south central Ethiopia revealed that children who were severely wasted were more likely to develop malarial attack [9]. Improving the nutritional status and the work status of mothers have a positive impact to reduce the prevalence of child undernutrition. The mother’s BMI, sex of child, age of a child, region of residence and weight of child at birth are the important biological and demographic determinants of malnutrition of children under five years in Ethiopia. The social-economic factors, such as household wealth and mother’s education, are also important determinants of undernutrition of a children under five years [10].

Most of the researchers in Ethiopia and abroad conducted a separate analysis of each anthropometric indicator, and identified the corresponding determinants [6, 8, 11, 12]. However, as the researchers’ knowledge concerned, there are very limited research papers conducted in Ethiopia that identified and estimates the determinants of under-five anthropometric indicators such as stunting, underweight and wasting by considering the association between them. Besides, even if the population in Ethiopia is not homogeneous in particular regarding to the region in terms of culture, language and soon, limited attention is given to take under consideration the cluster effect. In regards to does not considering the cluster effect, the information determinants of undernutrition status of a child within and between regions (cluster) will be insufficiently considered as children from the same region more likely to have similar undernutrition status as compared to children from different regions. As a result, implementing a multilevel model which also account the hierarchical nature is necessary. Therefore, this study aimed to identify and estimate the effects of important determinants of anthropometric indicators by taking into account their association and clustering effects.

## Methods

### Data source

The data for this study was obtained from 2019 Ethiopia Mini Demographic and Health Survey (EMDHS) [13] involved a total of 5027 under-five children. The 2019 EDHS was implemented by the Ethiopian Public Health Institute (EPHI), in partnership with the Federal Ministry of Health (FMoH) and the Central Statistical Agency (CSA), under the overall guidance of the Technical

Working Group (TWG). It is the second EMDHS and the fifth DHS implemented in Ethiopia. Funding for the 2019 EMDHS was provided by the United Nations Children’s Fund (UNICEF), the World Bank, and the United States Agency for International Development (USAID). It is designed to provide a data for measuring the up-to-date progress of the health sector goals such as estimate early childhood mortality and assessing the nutritional status of children under age five [13].

### Inclusion/exclusion criteria

The inclusion criteria were age below five years and completed relevant forms about the personal information and clinical signs. Hence, children who had not completed all relevant information or aged greater than or equal to five years were excluded.

### Sampling and data collection procedure

The EMDHS data collection lasted from March to June 2019, based on a nationally representative sample that provided estimates at the national and regional levels and for urban and rural areas. The sampling frame used for the 2019 EMDHS is a frame of all census enumeration areas (EAs) created for the 2019 Ethiopia Population and Housing Census (EPHC) and conducted by the CSA. Ethiopia is divided into nine geographical regions and two administrative cities for better administrative purpose. The sample for the 2019 EMDHS was designed to provide estimates of key indicators for each of the nine regions and the two administrative cities [13].

The sample was stratified and selected in two stages, to which enumeration areas (EAs) were the sampling units for the first stage. In the first stage, a total of 305 EAs were selected with probability proportional to EA size and with independent selection in each sampling stratum.

The resulting lists of households served as a sampling frame for the selection of households in the second stage. In the second stage of selection, households per EA were selected with an equal probability systematic selection from the newly created household listing. Based on the women’s questionnaire, in all the selected households, data of children along with its complete anthropometric indicators was considered [13].

### Variables

#### Dependent variables

The anthropometric indicators stunting, underweight, and wasting were the three dependent variables considered in this study. The stunting, underweight and wasting status of a child is computed from height-for-age z-score (HAZ), weight-for-age z-score (WAZ), and a weight-for-height z-score (WHZ), respectively, using the 2006 WHO child growth standards. Children who have a HAZ, WHZ and WHZ value below two are defined as having stunting, underweight and wasting respectively [14, 15]. After the z-score for each child is calculated, the dependent variables was recoded into binary outcomes as: stunted (0 = No if HAZ ≥ -2 and 1 = Yes if HAZ *<* - 2), wasted (0 = No if WHZ ≥ -2 and 1 = Yes if WHZ *<* -2), and underweight (0 = No if WAZ ≥ -2 and 1 = Yes if WAZ *<* - 2) according to WHO child growth standards [15].

#### Independent variables

The selection of independent variables was carried out based on previous research with regard to factors affecting children’s undernutrition status [5, 14, 15] and other important variables in the 2019 EMDHS. These variables are constructed by creating categories from naturally continuous and discrete variables. In total seventeen independent variables obtained from children and their respective households were considered (see Table 1).

**Table 1:** Independent variables description and frequency distribution

Variables	Categories (Codes)	n (%)
Sex of child	Male (1)	2576 (51.2)
	Female (2)	2451 (48.8)
Age of child	0–5 (0)	548 (10.9)
	6–11 (1)	489 (9.7)
	12–23 (2)	957 (19.0)
	24–35 (3)	1028 (20.4)
	36–47 (4)	9975 (19.4)
	48–59 (5)	1030 (20.5)
Birth Order	1st (0)	1048 (20.8)
	2–3 (1)	1692 (33.7)
	4–5 (2)	1151 (22.9)
	6 and more (3)	1136 (22.6)
Multiple birth	Single birth (0)	4918 (97.8)
	1st of multiple (1)	61 (1.2)
	2nd of multiple (2)	48 (1.0)
Residence	Urban (1)	1157 (23.0)
	Rural (2)	3870 (77.0)
Region	Tigray (1)	429 (8.5)
	Afar (2)	561 (11.2)
	Amhara (3)	459 (9.1)
	Oromia (4)	638 (12.7)
	Somali (5)	532 (10.6)
	Benishangul (6)	452 (9.0)
	SNNPR (7)	591 (11.8)
	Gambela (8)	381 (7.6)
	Harari (9)	380 (7.6)
	Addis Ababa (10)	257 (5.1)
	Dire Dawa (11)	347 (6.9)
Source of drinking water	unimproved source (0)	2042 (40.6)
	Improved source (1)	2986 (59.4)
Number of children under-five years in the household	1 (0)	1854 (36.9)
	2 (1)	2301 (45.8)
	3 or more (2)	872 (17.3)
Husband/partner’s education level	No education (0)	2766 (55.0)
	Primary (1)	1569 (31.2)
	Secondary and above (2)	692 (13.8)
Age of mothers at first birth	*<* 20 (0)	3185 (63.4)
	20–34 (1)	1827 (36.3)
	35–49 (2)	15 (0.3)
Mother’s current marital status	Married (1)	4745 (94.4)
	Widowed (2)	140 (2.8)
	Divorced (3)	142 (2.8)
Number of living children	1–4	3350 (66.6)
	5–9	1618 (32.2)
	10 and more	59 (1.2)
Place of delivery	Home (1)	2481 (49.4)
	Health sector (2)	2546 (50.6)
Breastfeeding	yes (1)	3134 (62.3)
	no (0)	1409 (28.0)
	Missing	484 (9.6)
Religion	Orthodox (1)	1461 (29.1)
	Protestant (2)	934 (18.6)
	Muslim (3)	2532 (50.4)
	Others (4)	100 (2.0)
Household size	1–4 (small) (0)	1368 (27.2)
	5–9 (medium) (1)	3300 (65.6)
	10 and more (Large) (2)	359 (7.1)
Household wealth index	Poorest (0)	1681 (33.4)
	Poorer (1)	883 (17.6)
	Middle (2)	706 (14.0)
	Richer (3)	654 (13.0)
	Richest (4)	1103 (21.9)

### Statistical analysis

#### Logistic regression

Logistic regression analysis was used to check the effect of independent variables on the categorical dependent variables [5]. Specifically, when a categorical dependent variable is dichotomous, binary logistic regression is used. The binary logistic regression model is considered only single response variable with binary outcome given other covariates [3]. Let **Y**_*i*_ = (*Y*_1*i*_*,Y*_2*i*_*,Y*_3*i*_) be a vector of binary responses indicating whether the *i*^*th*^ child is underweight (*Y*_1*i*_ = 1), stunting (*Y*_2*i*_ = 1) and wasting (*Y*_3*i*_ = 1) while the **X**_*i*_ the vector of covariates for the i^*th*^ child. Therefore, the binary logistic regression model is given as [3, 5]:

1 $$logit\left[P\left({Y}_{ij}=1/X\right)\right]={\beta}_{1j}{x}_{i1}+{\beta}_{2j}{x}_{i2}+\dots +{\beta}_{pj}{x}_{ip}={X\beta}_j,j=1,2,3$$

where *β*_*j*_ is a vector of coefficients of covariates, tells us the effect of covariates on the dependent variables. *P*(*Y*_*ij*_ = 1*/X*) is the probability of the *i*^*th*^ child being underweight *P*(*Y*_*i*1_), stunting *P*(*Y*_*i*2_) and/or wasting *P*(*Y*_*i*3_) given other covariates X. These probabilities can be calculated as [16]:

2 $$P\left({Y}_{ij}=1\right)=\frac{e^{\beta_{1j}{x}_{i1}+{\beta}_{2j}{x}_{i2}+\cdots {\beta}_{pj}{x}_{ip}}}{1+{e}^{\beta_{ij}{x}_{i1}}+{\beta}_{2j}{x}_{i2}+\cdots +{\beta}_{pj}{x}_{ip}}=\frac{e^{{X\beta}_j}}{1+{e}^{{X\beta}_j}}$$

The effect of covariates on the dependent variables (Y_*ij*_) is commonly interpreted using odds ratio [5]. Odds ratio (OR_*j*_) is the ratio of two odds and defined as:

3 $${OR}_j=\frac{Odds_{j1}}{Odds_{j1}}=\frac{\pi_j\left({x}_1\right)/\left(1-{\pi}_j\left({x}_1\right)\right)}{\pi_j\left({x}_2\right)/\left(1-{\pi}_j\left({x}_2\right)\right)}$$

Where, *π*_*j*(*X*1)_ and *π*_*j*(*X*2)_ are the probability of a child being underweight (j = 1), stunting (j = 2) and wasting (j = 3) for the values of variable X are x1 and x2 respectively. The odds ratio departure from 1 indicates the extent of dependence between variables.

### Multilevel multivariate logistic regression

A separate analysis of anthropomorphic indicators underweight, stunting and wasting on under five children are conducted in numerous research papers [2, 4, 6, 8]. In such a case applying logistic regression analysis to estimate the effect of covariates on a dependent variable is sufficient. However, implementing a separate analysis would ignore the dependency between the anthropomorphic indicators. To take under consideration of the correlation between the anthropometric indicators and the estimates of effects of covariates, multivariate logistic regression model is a more plausible alternative [5]. This statistical model serve to model more than one categorical outcome of interest at a time and assess their association given that of other covariates [5, 17].

However, still applying multivariate logistic regression model is not sufficient to estimate the effect of covariates on anthropometric indicators for a country consists heterogeneous population like Ethiopia. As the EMDHS data collected from children living in different region in Ethiopia, the likelihood to have a clustering effect is very high. Thus, if there is clustering effect in the dataset there will be a mess results. The clustering effect was checked using intra class correlation coefficient (ICC) [18] and median odds ratio (MOR) [19]. The ICC can be calculated as:

4 $$ICC=\frac{{\hat{\sigma}}_r^2}{{\hat{\sigma}}^2+{\hat{\sigma}}_r^2}$$

where *σ*ˆ_*r*_^2^ and *σ*ˆ^2^ are the estimated cluster variance (regarding to region) and residual variance, respectively [18]. The calculated ICC of anthropometric indicator stunting, underweight and wasting were 56.2, 52.5, and 53.2% respectively, which is high. This indicates that there is a clustering effect and strongly suggestive that there is within-group variability that would benefit from a cluster effect because of region. In this sense, a high ICC indicates high similarity between children anthropometric indicators’ from the same region. On the other hand, the estimated clustering variance regarding to region on stunting (*σ*ˆ_*rs*_^2^), underweight (*σ*ˆ_*ru*_^2^) and wasting (*σ*ˆ_*r*_^2^) found to be significant (*p*-value*<* 0.05) in the model indicates that there is region effect in the model, see Table 2.

**Table 2:** The association between covariates and each anthropometric indicator, EMDHS 2019

Covariates	Stunting		*p*-value	Underweight		*p*-value	Wasting		*p*-value
	Yes(%)	No(%)		Yes(%)	No(%)		Yes(%)	No(%)	
Sex of child									
male	976 (54.0)	1600	0.002	653 (55.7)	1923 (49.9)	0.000	277 (60.5)	2299 (50.3)	0.000
female	831 (46.0)	(49.7) 1620 (50.3)		519 (44.3)	1932 (50.1)		181 (39.5)	2270 (49.7)	
Age of child									
0–5	87 (4.8)	461(14.3)	0.920	61 (5.2)	487 (12.6)	0.000	64 (14.0)	484 (10.6)	0.372
6–11	112 (6.2)	377 (11.7)		84 (7.2)	405 (10.5)		42 (9.2)	447 (9.8)	
12–23	329 (18.2)	628 (19.5)		203 (17.3)	754 (19.6)		87 (19.0)	870 (19.0)	
24–35	463 (25.6)	565 (17.5)		298 (25.4)	730 (18.9)		91 (19.9)	937 (20.5)	
36–47	403 (22.3)	572 (17.8)		242 (20.6)	733 (19.0)		81 (17.7)	894 (19.6)	
48–59	413 (22.9)	617 (19.2)		284 (24.2)	746 (19.4)		93 (20.3)	937 (20.5)	
Number of children (*<* 5 *yrs*)									
one	611 (33.8)	1243 (38.6)	0.001	392 (33.4)	1462 (37.9)	0.003	138 (30.1)	1716 (37.6)	0.001
two	884 (48.9)	1417 (44.0)		544 (46.4)	1757 (45.6)		217 (47.4)	2084 (45.6)	
three and more	312 (17.3)	560 (17.4)		236 (20.1)	636 (16.5)		103 (22.5)	769 (16.8)	
Residence									
Urban	280 (15.5)	877 (27.2)	0.000	182 (15.5)	975 (25.3)	0.000	77 (16.8)	1080 (23.6)	0.000
Rural	1527 (84.5)	2343 (72.8)		990 (84.5)	2880 (74.7)		381 (83.2)	3489 (76.4)	
Region									
Tigray	220 (12.2)	209 (6.5)	0.000	134 (11.4)	295 (7.7)	0.000	37 (8.1)	392 (8.6)	0.000
Afar	244 (13.5)	317 (9.8)		180 (15.4)	381 (9.9)		81 (17.7)	480 (10.5)	
Amhara	201 (11.1)	258 (8.0)		126 (10.8)	333 (8.6)		39 (8.5)	420 (9.2)	
Oromia	237 (13.1)	401 (12.5)		108 (9.2)	530 (13.7)		31 (6.8)	607 (13.3)	
Somali	167 (9.2)	365 (11.3)		163 (13.9)	369 (9.6)		117 (25.5)	415 (9.1)	
Benshangul	185 (10.2)	267 (8.3)		132 (11.3)	320 (8.3)		29 (6.3)	423 (9.3)	
SNNPR	222 (12.3)	369 (11.5)		119 (10.2)	472 (12.2)		35 (7.6)	556 (12.2)	
Gambela	73 (4.0)	308 (9.6)		66 (5.6)	315 (8.2)		42 (9.2)	339 (7.4)	
Harari	128 (7.1)	252 (7.8)		68 (5.8)	312 (8.1)		17 (3.7)	363 (7.9)	
Addis Ababa	37 (2.0)	220 (6.8)		14 (1.2)	243 (6.3)		6 (1.3)	251 (5.5)	
Dire Dawa	93 (5.1)	254 (7.9)		62 (5.3)	285 (7.4)		24 (5.2)	323 (7.1)	
Source of drinking water									
unimproved	796 (44.1)	1246 (38.7)	0.000	565 (48.2)	1477 (38.3)	0.000	252 (55.0)	1790 (39.2)	0.000
improved	1011 (55.9)	1974 (61.3)		607 (51.8)	2378 (61.7)		206 (45.0)	2779 (60.8)	
Breast feeding									
No	544 (33.5)	865 (29.6)	0.004	332 (31.4)	1077 (30.9)	0.398	132 (30.7)	1277 (31.0)	0.465
Yes	1080 (66.5)	2054 (70.4)		726 (68.6)	2708 (69.1)		298 (69.3)	2836 (69.0)	
Birth order									
1st	335 (18.5)	713 (22.1)	0.000	218 (18.6)	830 (21.5)	0.000	86 (18.8)	962 (21.1)	0.088
2–3	579 (32.0)	1113 (34.6)		358 (30.5)	1334 (34.6)		138 (30.1)	1554 (34.0)	
4–5	445 (24.6)	706 (21.9)		286 (24.4)	865 (22.4)		115 (25.1)	1036 (22.7)	
6 and more	448 (24.8)	688 (21.4)		310 (26.5)	826 (21.4)		119 (26.0)	1017 (22.3)	
Multiple birth									
Single birth	1749 (96.8)	3169 (98.4)	0.000	1132 (96.6)	3786 (98.2)	0.001	442 (96.5)	4476 (98.0)	0.126
1st of multiple	30 (1.7)	31 (1.0)		18 (1.5)	43 (1.1)		9 (2.0)	52 (1.1)	
2nd of multiple	28 (1.5)	20 (0.6)		22 (1.9)	26 (0.7)		7 (1.5)	41 (0.9)	
Religion									
Orthodox	556 (30.8)	905 (28.1)	0.000	341 (29.1)	1120 (29.1)	0.000	107 (23.4) 60	1354 (29.6)	0.000
Protestant	277 (15.3)	657 (20.4)		152 (13.0)	782 (20.3)		(13.1)	874 (19.1)	
Muslim	948 (52.5)	1584 (49.2)		654 (55.8)	1878 (48.7)		277 (60.5)	2255 (49.4)	
Others	26 (1.4)	74 (2.3)		25 (2.1)	75 (1.9)		14 (3.1)	86 (1.9)	
Mothers’ Age at 1st birth									
*<* 20	1202 (66.5)	1983 (61.6)	0.001	792 (67.6)	2393 (62.1)	0.001	308 (67.2)	2877 (63.0)	0.106
20–34	603 (33.4)	1224 (38.0)		379 (32.3)	1448 (37.48)		150 (32.8)	1677 (36.7)	
35–49	2 (0.1)	13 (0.4)		1 (0.1)	14 (0.4)		0 (0.0)	15 (0.3)	
Place of delivery									
Home	960 (53.1)	1521 (47.2)	0.000	676 (57.7)	1805 (46.8)	0.000	283 (61.8)	2198 (48.1)	0.000
Health sector	847 (46.9)	847 (1699)		496 (42.3)	2050 (53.2)		175 (38.2)	2371 (51.9)	
Total children in the household									
1–4	1146 (63.4)	2204 (68.4)	0.001	699 (59.6)	2651 (68.8)	0.000	272 (59.4)	3078 (67.4)	0.003
5–9	639 (35.4)	979 (30.4)		458 (39.1)	1160 (30.1)		180 (39.3)	1438 (31.5)	
10 and more	22 (1.2)	37 (1.1)		15 (1.3)	44 (1.1)		6 (1.3)	53 (1.2)	
Husband/partner’s education level									
No education	1123 (62.1)	1643 (51.0)	0.000	781 (66.6)	1985 (51.5)	0.000	317 (69.2)	2449 (53.6)	0.000
Primary	554 (30.7)	1015 (31.5)		325 (27.7)	1244 (32.3)		108 (23.6)	1461 (32.0)	
Secondary and higher	130 (7.2)	562 (17.5)		66 (5.6)	626 (16.2)		33 (7.2)	659 (14.4)	
Household size									
1–4	457 (25.3)	911 (28.3)	0.063	275 (23.5)	1093 (28.4)	0.001	105 (22.9)	1263 (27.6)	0.003
5–9	1222 (67.6)	2078 (64.5)		796 (67.9)	2504 (65.0)		305 (66.6)	2995 (65.6)	
10 and more	128 (7.1)	231 (7.2)		101 (8.6)	258 (6.7)		48 (10.5)	311 (6.8)	
Wealth index									
poorest	674 (37.3)	1007 (31.3)	0.000	519 (44.3)	1162 (30.1)	0.000	246 (53.7)	1435 (31.4)	0.000
poorer	360 (19.9)	523 (16.2)		235 (20.1)	648 (16.8)		74 (16.2)	809 (17.7)	
middle	293 (16.2)	413 (12.8)		161 (13.7)	545 (14.1)		43 (9.4)	663 (14.5)	
richer	239 (13.2)	415 (12.9)		116 (9.9)	538 (14.0)		40 (8.7)	614 (13.4)	
richest	241 (13.3)	862 (26.8)		141 (12.0)	962 (25.0)		55 (12.0)	1048 (22.9)	
Marital Status									
Married	1720 (95.2)	3025 (93.9)	0.108	1109 (94.6)	3636 (94.3)	0.752	432 (94.3)	4313 (94.4)	0.484
Divorced	39 (2.2)	101 (3.1)		29 (2.5)	111 (2.9)		10 (2.2)	130 (2.8)	
Widowed	48 (2.7)	94 (2.9)		34 (2.9)	108 (2.8)		16 (3.5)	126 (2.58)	

The MOR is defined as: $\mathrm{MOR}=exp\left\{0.6745\sqrt{2\mathrm\sigma_{\mathrm r}^2}\right\}$ [5, 18]. The MOR value stunting (1.97), underweight (1.74), and wasting (1.76) are differ from one. This indicates that there is significant clustering variation. To take into account the cluster effect because of region, a multilevel multivariate logistic regression model was applied because the traditional model can not remove the cluster effect.

**Fig. 1:**
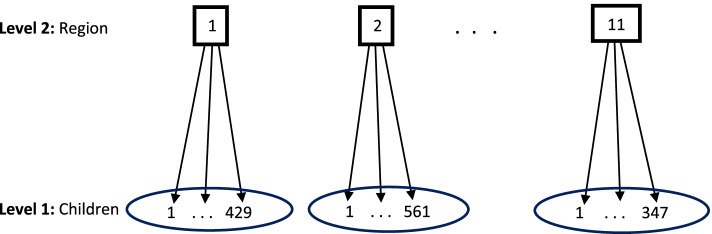
Multilevel structure of children anthropometric indices at level 1, nested within region at level 2

A multilevel structure of children anthropometric indices at level 1 nested within region at level 2 revealed in Fig. 1. Ethiopia consists eleven region including two administrative cities (see Table 1). The EDMHS data collected from each of the region. The three anthropometric indicators were measured from each child in the region.

Once the data arrangement was accomplished using SPSS 23, all the statistical analysis was done using R software VGAM and glmer package.

### Ethics approval

EMDHS Program granted permission to download and use the data for this study after being registered and submitting a request with briefly stated objectives of the study. The Institution Review Board approved procedures for DHS public use data sets that do not in any way allow respondents, households, or sample communities to be identified. There are no names of individuals or household addresses in the data files. The detail of the ethical issues has been published in the 2019 EMDHS final report, which can be accessed at:http://www.dhsprogram.com/publications

## Results

Among 5027 children considered in the study, the prevalence of stunted, underweight and wasted children were 1816 (36.0%, 1177 (23.3%), and 460 (9.1%) respectively (see Table 3). The independent variables’ description with frequency distribution were depicted in Table 1. More than half of the children (51.2%) were males and 77.0% lived in rural area. The majority of husbands/partners (55.0%) were not educated and 63.4% of mothers were aged below 20 at their first birth. Whereas 40.6% of the children were from household who have unimproved source of drinking water and 33.4% of the children were from household with poorest wealth index. The majority of the household (66.6%) had a total number of children between one and four, while the majority of the household (45.8%) had two under five children. Nearly half (49.4%) of the children were born at home and 62.3% of the children were feed breast milk. The household size of the majority households (65.6%) were between five and nine ( medium ).

**Table 3:** Dependent variables description and frequency distribution

Variables	Categories (codes)	n (%)
Stunting	yes (1)	1816 (36.0)
	no (0)	3234 (64.0)
underweight	yes (1)	1177 (23.3)
	no (0)	3873 (76.7)
wasting	yes (1)	460 (9.1)
	no (0)	4590 (90.9)

Most of the children are exposed to one or more anthropometric indicators. The frequency distribution of children with one or more anthropometric indicators were revealed in Table 4. 767 children had stunting, underweight and wasting. Whereas, 180 children had both underweight and stunting; 860 children had both stunting and wasting.

**Table 4:** The joint frequency distribution of stunting, wasting, and underweight

				Underweight		Total
				yes	no	
Stunting	yes	Wasted	yes	767	860	1627
			no	180	0	180
	no	Wasted	yes	154	124	278
			no	71	2871	2942
Total				1172	3855	5027

The frequency distribution of children within each anthropometric indicator in Table 4 can also be explained in Table 5. In Table 5 the undernutrition status of children were categorized into eight categories as nourished; wasting only; underweight only; stunting and wasting; stunting and underweight; underweight and wasting; and finally stunting, wasting and underweight. The proportion of children with stunting and underweight (17.1%) was the highest as compared to the proportion of children with other categories of undernutrition. Among 5027 children in this study, the total number of nourished children was 2871 (57.1%). This indicates that the total number of undernutrition children, i.e, children with at least one of the anthropometric indicator, is equal to 2156 (42.9%) {100 − 57*.*1%}.The pairwise dependency between stunting, underweight and wasting using odds ratio (OR) was revealed in Table 6. The OR values of the dependency between stunting and underweight; stunting and wasting; and underweight and wasting were 14.66, 1.17 and 11.99 respectively. The OR value deviated from one indicates that there is a dependency between the two anthropometric indicators. Thus, there is a dependency between stunting and underweight (OR = 14.66) and underweight and wasting (OR = 11.99).

**Table 5:** Cross classification of undernutrition category and corresponding frequency distribution

Undernutrition category	frequency (%)
Nourished	2871 (57.1)
Wasting only	124 (2.5)
Underweight only	71 (1.4)
Stunting and Wasting	860 (17.1)
Stunting and Underweight	180 (3.6)
Underweight and Wasting	154 (3.0)
Stunting, Wasting, and Underweight	767 (15.3)

**Table 6:** Pairwise dependency between anthropometric indicators stunting, underweight and wasting using OR

	Stunting, Underweight	Stunting, Wasting	Underweight, Wasting
OR	14.66	1.17	11.99

The bivariate association between anthropometric indicators (stunting, underweight and wasting) and each covaraite were depicted using a chi-square test statistics in Table 2. The sex of child, number of children aged under five, residence, region, source of drinking water, breast feeding, birth order, multiple birth, religion, mothers’ age at first birth, place of delivery, total number of children in the household, husband/partner’s education level and wealth index were independently associated with stunting (*p*-value*<*0.05). The number of male children with stunting 976 (54.0%) was higher than female. The majority of stunted children were from rural areas (84.5%) and from mothers’ below 20 years at first birth (66.5%). The highest proportion of stunted children were from the poorest household (37.3%) and from mothers delivered children at home (53.1%).

Both underweight and wasting of children were independently associated (*p*-value *<*0.05) with covariates such as sex of children, number of under five children in the household, residence, region, source of drinking water, religion, place of delivery, total children in the household and husband/partner’s education level. As compared to female children, the proportion of underweight (55.7%) and wasting (60.5%) of male children were higher. The highest prevalence of underweight (44.3%) and wasted (53.7%) children were from poorest household. Similarly, the majority of underweight (67.6%) and wasted (67.2%) children were from mothers aged below 20 years. The number of underweight and wasted children from protestant and orthodox religions were lower than underweight and wasted children from Muslim religion.

The effect of covariates on stunting, underweight, and wasting were estimated using multilevel multivariate logistic regression model in Table 7. In this method of statistical analysis possible dependency between the anthropometric indicators and the clustering effects due to region were taken into consideration. The pairwise dependency between underweight and stunting; underweight and wasting; and stunting and wasting given other covariates and clustering effect (regarding to region) using OR were 13.490, 15.220 and 1.110 respectively. The OR values for a dependency between underweight and stunting (13.490); and underweight and wasting (15.220) were quite differ from one (*P*-value*<* 0.05), indicates statistically significant dependency. The OR value for a dependency between stunting and wasting was 1.110 (*p*-value = 0.169 *>* 0.05) which is closer to one indicates statistically lack of dependency between stunting and wasting. The estimated cluster variance due to region given other covariates of stunting, underweight and wasting were 3.890 (*σ*ˆ_*rs*_^2),^ 3.360 (*σ*ˆ_*ru*_^2)^ and 3.450 (*σ*ˆ_*rw*_^2)^ respectively. The *p*-values of cluster variance for each of the anthropometric indicators less than 0.05 shows a significant clustering effect regarding the region.

**Table 7:** Parameter estimations of effects of covariates on stunting, underweight, and wasting using multilevel multivariate logistic regression model

Variables	Stunting		Underweight		Wasting	
	est.(sd.err)	OR (95% CI)	est.(sd.err)	OR (95% CI)	est.(sd.err)	OR (95% CI)
Intercept	−1.606 (0.313)	0.201(0.109,0.370)	−0.872(0.353)	0.418(0.209,0.835)	−0.300(0.048)	0.741(0.291,1.882)
Sex						
male(^a^)	0.000		0.000		0.000	
female	−0.212(0.065)	0.809(0.712,0.920)	−0.299(0.073)	0.741(0.642,0.856)	−0.446(0.105)	0.640(0.521,0.786)
Age of child						
0–5 (^a^)	0.000		0.000		0.000	
6-11 t	0.455(0.162)	1.577(1.148,2.166)	0.529(0.184)	1.697(1.184,2.433)	−0.303(0.211)	0.739(0.488,1.118)
12–23	1.134(0.139)	3.108(2.366,4.084)	0.897(0.160)	2.452(1.791,3.359)	−0.257(0.180)	0.773(0.543,1.100)
24–25	1.578(0.139)	4.845(3.688,6.364)	1.277(0.158)	3.587(2.630,4.891)	−0.358(0.182)	0.699(0.489,0.999)
36–47	1.446(0.143)	4.245(3.210,5.614)	1.101(0.163)	−3.008(2.187, 4.137)	−0.367(0.188)	0.693(0.479,1.000)
48–59	1.307(0.150)	3.696(2.755, 4.957)	1.131(0.169)	3.100(2.224,4.321)	−0.381(0.200)	0.684(0.462,1.010)
No. of children (*<* 5 yr)						
one(^a^)	0.000		0.000		0.000	
two	0.170(0.084)	1.185(1.006, 1.396)	−0.064(0.094)	0.938(0.780, 1.128)	0.056(0.137)	1.058(0.808,1.384)
three or more	0.074(0.116)	1.0765(0.858,1.351)	−0.032(0.127)	0.968(0.754, 1.242)	−0.026(0.178)	0.975(0.687,1.382)
Residence						
Rural(^a^)	0.000		0.000		0.000	
Urban	0.105(0.121)	1.111(0.876,1.409)	−0.286(0.138)	0.751(0.573,0.984)	−0.411(0.191)	0.663(0.456,0.965)
Source of drinking water						
Unimproved(^a^)	0.000		0.000		0.000	
Improved	−0.008(0.073)	0.992(0.859,1.146)	−0.065(0.081)	0.937(0.800,1.098)	−0.223(0.114)	0.800(0.640,1.002)
Breast Feeding						
No(^a^)	0.000		0.000		0.000	
Yes	0.082(0.076)	1.085(0.935,1.259)	0.210(0.085)	1.233(1.043,1.458)	0.0105(0.123)	1.011(0.794,1.286)
Birth Order						
1st (^a^)	0.000		0.000		0.000	
2–3	0.032(0.102)	1.033(0.845,1.261)	−0.120(0.115)	0.887(0.709,1.111)	−0.232(0.165)	0.793(0.575,1.100)
4–5	0.145(0.137)	1.156(0.884,1.512)	−0.311(0.156)	0.732(0.540,0.994)	−0.427(0.224)	0.652(0.421,1.011)
6 and more	0.234(0.174)	1.263(0.899,1.775)	−0.384(0.194)	0.681(0.466,0.996)	−0.543(0.276)	0.581(0.338,0.998)
Multiple Birth						
Single birth(^a^)	0.000		0.000		0.000	
1st of multiple	0.547(0.148)	1.727(1.292,2.310)	0.508(0.149)	1.663(1.242,2.225)	0.330(0.197)	1.391(0.946,2.044)
2nd of multiple	0.547(0.148)	1.727(1.292,2.310)	0.508(0.149)	1.663(1.242,2.225)	0.330(0.197)	1.391(0.946,2.044)
Religion						
Orthodox(^a^)	0.000		0.000		0.000	
Protestant	−0.568(0.105)	0.567(0.462,0.696)	−0.579(0.123)	0.560(0.440,0.713)	−0.268(0.184)	0.765(0.533,1.096)
Muslim	−0.195(0.083)	0.823(0.700,0.968)	−0.100(0.092)	0.905(0.755,1.083)	0.079(0.136)	1.082(0.829,1.412)
Others	−0.819(0.253)	0.441(0.268,0.723)	−0.225(0.263)	0.798(0.477,1.336)	0.446(0.322)	1.561(0.830,2.935)
Mother’s age at 1st birth						
*<* 20 (^a^)	0.000		0.000		0.000	
20–34	−0.067(0.072)	0.935(0.812,1.077)	−0.113(0.081)	0.893(0.763,1.046)	−0.145(0.115)	0.865(0.691,1.083)
35–49	−0.659(0.785)	0.517(0.111,2.409)	−0.710(1.018)	0.491(0.067,3.611)	−15.590(0.206)	0.001(0.000,0.125)
Mother’s current marital status						
Married (^a^)	0.000		0.000		0.000	
Divorced	−0.010(0.214)	0.990(0.651,1.507)	0.126(0.233)	1.134(0.717,1.792)	0.064(0.343)	1.067(0.544,2.088)
Widowed	−0.209(0.214)	0.811(0.533,1.235)	0.017(0.229)	1.017(0.650,1.592)	0.340(0.294)	1.405(0.790,2.499)
Place of delivery						
Health Sector(^a^)	0.000		0.000		0.000	
Home	0.172(0.078)	1.187(1.019,1.383)	0.036(0.086)	1.037(0.875,1.229)	−0.072(1.250)	0.930(0.728,1.189)
Total children in the HH						
1–4(^a^)	0.000		0.000		0.000	
5–9	−0.295(0.124)	0.744(0.584,0.948)	0.173(0.138)	1.189(0.907,1.559)	0.283(0.198)	1.328(0.901,1.956)
10 and more	−0.617(0.352)	0.539(0.270,1.076)	−0.379(0.406)	0.685(0.309,1.516)	0.168(0.506)	1.183(0.438,3.190)
Education levels of women						
No education(^a^)	0.000		0.000		0.000	
Primary	−0.080(0.082)	0.923(0.785,1.084)	−0.191(0.092)	0.827(0.690,0.991)	−0.311(0.137)	0.733(0.560,0.959)
Secondary and above	−0.702(0.135)	0.496(0.380,0.646)	−0.845(0.168)	0.430(0.309,0.600)	−0.546(0.235)	0.580(0.366,0.919)
Household Size						
Small (1–4)(^a^)	0.000		0.000		0.000	
Medium (5–9)	0.004(0.096)	1.004(0.832,1.211)	0.077(0.109)	1.080(0.872,1.334)	0.157(0.156)	1.170(0.861,1.589)
10 and more	0.015(0.166)	1.015(0.733,1.406)	0.270(0.180)	1.310(0.921,1.864)	0.421(0.242)	1.523(0.948,2.447)
Wealth Index						
Poorest(^a^)	0.000		0.000		0.000	
Poorer	0.058(0.097)	1.059(0.877,1.280)	−0.146(0.104)	0.864(0.704,1.060)	−0.465(0.153)	0.628(0.466,0.847)
Middle	0.118(0.109)	1.125(0.910,1.392)	−0.283(0.122)	0.754(0.594,0.957)	−0.700(0.192)	0.496(0.341,0.723)
Richer	−0.052(0.117)	0.949(0.755,1.195)	−0.558(0.138)	0.572(0.437,0.749)	−0.621(0.201)	0.538(0.362,0.798)
Richest	−0.723(0.149)	0.485(0.362,0.651)	−1.046(0.174)	0.351(0.250,0.494)	−0.961(0.247)	0.383(0.236,0.621)
**Random effect**	*σ*^ˆ^_*rs*_^2^ (sd.err)	*P*-value	*σ*ˆ_*ru*_^2^ (sd.err)	*P*-value	*σ*ˆ_*rw*_^2^ (sd.err)	*P*-value
Region	3.890 (0.158)	0.001	3.360 (0.122)	0.015	3.450 (0.544)	0.032
**Association**	**Odds Ratio(OR)**		***P*-value**			
Underweight and Stunting	13.490		0.0001			
Underweight and Wasting	15.220		0.0001			
Stunting and wasting	1.110		0.169			

Key: ^a^= Reference group; *OR* Odds Ratio, *CI* Confidence Interval, *est*. estimate, *sd.err* standard error

The estimated 95% CI’s of OR for a covariate not includes one indicates that a covariate has significant effect on the corresponding anthropometric indicator. The covariates such as sex of child, age, total children in the household, multiple birth, religion, place of delivery, husband/partner’s education level and wealth index have a significant effect on stunting. The estimated odds of female child to be stunted was 0.81 (OR=0.81) times the estimated odds of male which suggests that female children had a higher risk of stunting than male. The estimated odds of children from protestant and Muslim being stunted were lower by 43.3% (OR=0.567) and 17.7 % (OR=0.823)of the estimated odds of children from orthodox religion respectively. Children whose place of delivery at home were more likely (OR=1.187) to be stunted than children whose place of delivery is at health sector. The estimated odds of children from household with secondary and above education level being stunted was 0.496 (OR=0.496) times the estimated odds of children from household with no education. This indicates that children from non-educated household were more likely being stunted than children from household with secondary and above education level. Whereas children from richest household were less likely to be stunted as compared to children from the poorest household (OR=0.485).The household wealth index, sex of child, residence, and husband/partner’s education level were common covariates that have a significant effect on both underweight and wasting of children.

The estimated odds of children from richest household to be underweight and wasting was 0.351 and 0.383 times the estimated odds of children from the poorest household respectively. The estimated odds of children from urban areas to be underweight and wasting were lower by 24.9% (OR = 0.751) and 33.7% (OR = 0.663) of estimated odds of children from rural area which indicates that children from urban areas are less likely to be underweight and wasted. Age of child and breast feeding have a significant effect on underweight whereas mothers’ age at first birth has also a significant effect on wasting.

## Discussion

This study was aimed to determine important covariates of anthropometric indicators stunting, underweight and wasting on under five children using multilevel multivariate logistic regression model based on a data from EDMHS 2019. The model is designed to evaluate the dependency between stunting, underweight and wasting as well as to estimate the clustering (region) effect given other covariates. Stunting, underweight and wasting are the three common anthropometric indicators used to measures the undernutrition status of a child computed from standardized height-for-age, weight-for-age and weight-for-height respectively [2, 4, 8]. Higher prevalence of malnutrition was noticed as compared to studies in Africa in particular, in Tanzania [2] and Uganda [4]. The prevalence of children with stunting (36.0%), underweight (23.3%), and wasting (9.1%) was higher as compared to the study findings based on 2016 EDHS in Ethiopia [5, 8] which violates the Millennium development goal and Ethiopian ministry of health plan. Many of the children were exposed to more than one undernutrition status. The proportion fo children with stunting, underweight and wasting was 15.3%, whereas the proportion of children with stunting and wasting was 17.1%. Few studies in Africa such as in Nigeria [5] and Iran [20] have been done on the dependency between the anthropometric indices. In this study the dependency between underweight and stunting; and underweight and wasting was statistically significant which was inline with a study reported based on 2018 NDHS data in Nigeria and Iran [5, 20], states that underweight of under five children significantly associated with both stunting and wasting. However, there was a lack of dependency between stunting and wasting.

The sex and age of child, total children in the household, religion, place of delivery, husband/partner’s education level and wealth index are important determinants that have a significant effect on stunting. The likelihood of a female child to stunted was lower than male which is supported by a findings in Tanzania [2] and Afar regional state of Ethiopia [6]. Children from household with no education were more likely to be stunted as compared to children from household with secondary and above educational level. Thus, educated household enables to provide better care of children. This was also in line with several studies on malnutrition in children aged under five in Ethiopia [3, 12] and abroad [2, 10] states that education of household is an important input to get logical skills on prenatal as well as postnatal children cares such as nutritional needs and health facilities. In Ethiopia most of the households has unplanned family size and hence the total number of children within the household is more than the economic status of the household. As a result, a child from household with higher number of children is more likely to be undernourished as compared to a child from household with lower number of children [1]. Thus, being familiar with family planning that would be compatible with the household economic status is advisable. In middle and low income countries such as Ethiopia wealth index is the most predominant factors of children undernutrition. A child belonging to the lower wealth index are more exposed to stunting as compared to a child belonging to the higher wealth index. This finding was comparable with the study in Uganda [4], which justifies that household socioeconomic status minimize the occurrence of stunting among children aged under five.

The study also revealed that place of residence, household wealth index, sex of child and education level are common significant determinants that affect both underweight and wasting. This was in line with study findings in Bangladesh based on data 2007 BDHS [1]. The risk of wasting was more prevalent on males than females which was also reported in [21]. In contrast, a study on children aged 36-60 months [8] reported that the likelihood of wasting significantly lower among male children than female children. Unlike a study in [1], a child from rural area had higher risk of being underweight and wasted as compared to urban area. This might be because of the fact that children in rural area belong to poor care in health facility, nutritional needs and lack of other infrastructure. The partners of the children in rural area don’t have enough awareness about how children needs to be grown. There is a scarcity even to fulfill basic necessity such as cloth, food and shelter. Most of under five children even moving in and out of home in naked and contact with mad and other unnecessary stuffs that might affect their health. Breast feeding and mothers age at first birth have also a significant effect on underweight and wasting respectively. The lower wealth index of the household highly associated with higher risk of underweight and wasting among under five children as it is also supported in [7].

It was also noticed that significant clustering effect on stunting, underweight and wasting due to region which indicates that the undernutrition status of children closer within the region and different between regions. This was consistent with a study in Nigeria [22] reported that the prevalence of child malnutrition varied significantly not only among different nations of the world but also in different regions of a country. The majority proportion of stunting (13.5%) and underweight (15.4%) was perceived in Afar region of Ethiopia as compared to other regions in the country. This might be because of that the region is desert and a place where pastoral communities are living [6, 23] which is not comfortable for planting crops that enables to be easily consumed by children.To end, as the prevalence of anthropometric indicators stunting, underweight and stunting is high, there is a need for policymakers and stakeholders to direct resources to reduce numerous impacts due to undernutrition by taken into under consideration the important biological, demographic and socioeconomic determinants that identified in the study.

## Conclusion

The prevalence of anthropometric indicators stunting, underweight and wasting in Ethiopia was increased. The children underweight has a significant dependency with both stunting and wasting. However, there was a lack of dependency between stunting and wasting. The sex of child, wealth index and education levels of a household are the common important determinants of stunting, underweight and wasting. The lower wealth index and education level of the household were highly associated with higher risk of stunting, underweight and wasting among under five children. The likelihood of a child from urban area being underweight and wasting was higher as compared to a child from rural area. The clustering effect on stunting, underweight and wasting due to region was statistically significant which indicates that the undernutrition status of children is more alike within the region and different between regions.

The authors would like to recommend if governmental and non-governmental stakeholders build short and long term food supplementary programs that enable to reduce the existence of child undernutrition and related adverse health effects by taking into account the important determinants.

## Data Availability

The datasets for generated analyses during the study is available in Ethiopian Demographic and Health Survey data if unique request sent via their site EMDHS 2019.

## Abbreviations

EMDHS: Ethiopian mini demographic and health survey
EDHS: Ethiopian demographic and health survey
BHDS: Bangladesh demographic and health survey
NDHS: Nigerian demographic and health survey
OR: Odds ratio
CI: Confidence interval
SNNPR: South Nations Nationalities and Peoples Representatives
ICC: Intra class correlation coefficient
MOR: Median odds ration
WHO: World health organization
